# Catheter-associated Hafnia alvei-induced Urosepsis

**DOI:** 10.7759/cureus.6471

**Published:** 2019-12-26

**Authors:** Keerthi Yarlagadda, Isha Shrimanker, Vinod K Nookala

**Affiliations:** 1 Internal Medicine, University of Pittsburgh Medical Center Pinnacle, Harrisburg, USA

**Keywords:** hafnia alvei, urosepsis, antibiotic resistance, catheterization

## Abstract

Hafnia alvei, belonging to the Enterobacteriaceae family, is a gram-negative, facultative anaerobe. The organism predominantly colonizes the gastrointestinal tract and, less often, the tissues, urine, and catheters. A 75-year-old male presented with a dry cough, fatigue, decreased appetite, intermittent disorientation, and difficulty ambulating. He had a history of self-catheterization due to urinary retention. He was scheduled to undergo transcatheter aortic valve replacement for enterococcal endocarditis. Physical examination was not significant for any changes. Urine analysis revealed positive leukocyte esterase, the presence of red and white blood cells, urine bacteria, and hyaline casts. The patient was started on empiric intravenous ceftriaxone. Urine culture grew Hafnia alvei and he was switched over to cefepime due to greater susceptibility. On day four, he started deteriorating clinically and was treated with four pressors but remained hypotensive and eventually became anuric. The patient developed septic shock with multiple organ dysfunction syndromes. Despite all measures, his clinical condition failed to improve, and he was continued with comfort measures only. The literature on Hafnia alvei-induced urosepsis is poor and fragmentary. Our patient showed resistance to most beta-lactam antibiotics, including cefuroxime, ceftriaxone, and ceftazidime, along with intermediate susceptibility to piperacillin/tazobactam and was managed with intravenous cefepime based on the sensitivity report. Inducible Bush group 1 beta-lactamase produced by Hafnia alvei is postulated to be responsible for antibiotic resistance. Physicians should remain vigilant of Hafnia alvei-induced urosepsis in patients with long-term catheterization initiating appropriate treatment.

## Introduction

Formerly known as Enterobacter hafniae or “paracolon” bacterium, Hafnia alvei (H. alvei) is a rod-shaped, gram-negative, facultative anaerobe, which is part of the Enterobacteriaceae family [1]. A taxonomic study was published on the genus Hafnia by Greipsson and Priest in 1983 [2]. It is an oxidase-negative, catalase-positive, and non-sporulating organism. H. alvei shows a similarity with Escherichia coli O157 on MacConkey agar since these organisms are D-sorbitol negative. It grows in culture media, which contains 2% to 5% sodium chloride, within a 4.9 to 8.25 pH range, and with an optimum temperature of 35°C [2].

This organism can be obtained from water, food such as milk and dairy products, meat, freshwater fish, as well as soil and sewage [2]. It is predominantly defined as an organism of the gastrointestinal tract and rarely considered to be pathogenic. In 1991, H. alvei was considered enteropathogenic for the first time [3]. It has been known to cause meningitis [4], pneumonia [5], wound infections [5], septicemia [6], and urinary tract infections [7-8].

In the past two decades, the systemic involvement of H. alvei has been looked into by a few studies [2]. There has been an association of the poultry industry with two H. alvei infection outbreaks [9]. Here, we present a case of an elderly male with a history of long-term catheterization and aortic valve disease, who developed H. alvei-induced urosepsis.

## Case presentation

**Figure 1:**
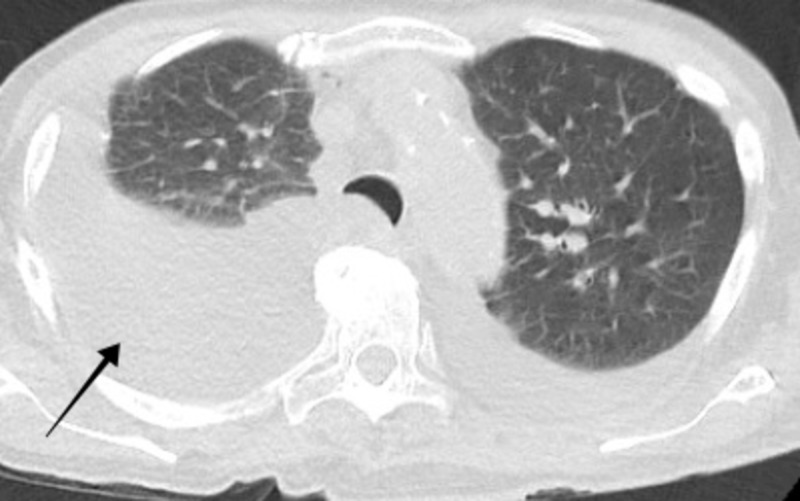
Computed tomography of the chest showed right-sided pleural effusion

**Figure 2:**
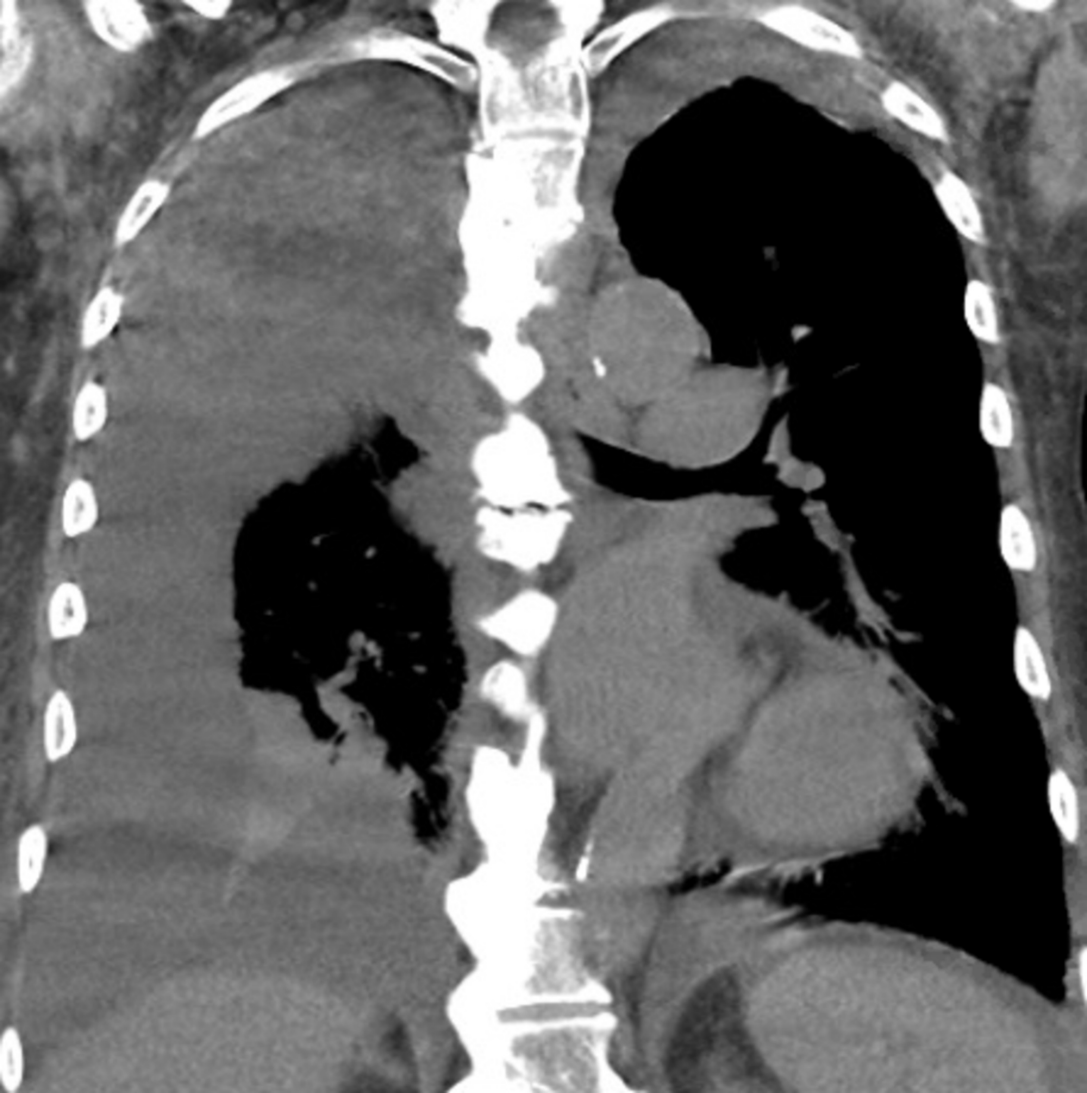
Computed tomography of the chest showed right-sided pleural effusion

A 75-year-old male presented to the emergency department with symptoms of dry cough, fatigue, decreased appetite, intermittent disorientation, and difficulty ambulating. He did not have complaints of fever, foul-smelling urine, hematuria, or abdominal pain. He had a history of mild aortic insufficiency with severe aortic stenosis and diastolic heart failure with an ejection fraction of 20%-25%. He was previously admitted for enterococcal endocarditis and was treated with intravenous (IV) antibiotics for a period of three months. He was scheduled to undergo transcatheter aortic valve replacement. For a period of one year, he used to self-catheterize approximately four times a day due to urinary retention present due to a neurogenic bladder. On admission, his pulse was 99 beats per minute, blood pressure 104/68 mm of Hg, oxygen saturation of 99% on room air and respiratory rate 21 breaths per minute. On physical exam, he had dry mucous membranes, was lethargic, and had decreased urine volume but dark in color. Workup revealed platelet count 90 K/ul (normal: 140 - 366 K/ul), troponin I 0.06 ng/ml (0.0 - 0.05 ng/ml), brain natriuretic peptide 3620 pg/ml (0 - 100 pg/mL), lactic acid 2.3 mmol/l (0.5 - 1.9 mmol/l), bilirubin 3.3 mg/dl (0.3 - 1.0 mg/dl), glucose 116 mg/dl (65 - 99 mg/dl), creatinine 1.4 mg/dl (0.7 - 1.3 mg/dl), and blood urea nitrogen 44 mg/dl (7 - 25 mg/dl). Computed tomography of the chest showed right-sided pleural effusion (Figures 1-2).

Urine analysis was suggestive of protein +1, urobilinogen +4, leukocyte esterase +2, red blood cells 6-10/hpf, white blood cells 16-30/hpf, urine bacteria +2, hyaline casts >50/lpf, and white blood cell clump present. The patient underwent ultrasound-guided thoracocentesis and approximately 1600 ml of fluid was drained out. He was started on empiric IV ceftriaxone. Urine culture revealed 10,000 - 49,000 cfu/ml of Hafnia alvei, growth was seen on tryptic soy agar, sheep blood agar, and MacConkey agar. The organism was resistant to most beta-lactam antibiotics, including ceftriaxone and ceftazidime, however, there was intermediate susceptibility to piperacillin/tazobactam. Blood culture was negative. He was then switched over to IV cefepime as per the sensitivity index (Table 1).

**Table 1:** Antibiotic susceptibility of H. alvei with minimum inhibitory concentration (MIC) MIC: minimum inhibitory concentration; R: Resistant; S: Susceptible; I: Intermediate

Antibiotic	MIC	Susceptibility
Ampicillin	>16	R
Cefepime	<8	S
Ceftazidime	>16	R
Ceftriaxone	>32	R
Cefuroxime	>16	R
Ertapenem	<0.5	S
Gentamicin	<2	S
Imipenem	<1	S
Levofloxacin	<2	S
Nitrofurantoin	<32	S
Piperacillin/Tazobactam	64	I
Tetracycline	<4	S
Trimethoprim + Sulfamethoxazole	<2/38	S

Repeat urine culture did not reveal any growth. On day four, the patient started deteriorating clinically with a blood pressure of 80/50 mm of Hg and urine output of 0.2 ml/kg/body weight. He was treated with four pressors but continued to be hypotensive and eventually became anuric. He was diagnosed with septic shock and multiple organ dysfunction syndromes. Since he did not show any signs of clinical improvement, he was continued with terminal measures focusing on his comfort.

## Discussion

H. alvei has been isolated from cases of hemolytic uremic syndrome, community-acquired urinary tract infection (UTI) [7,10], and nosocomial urosepsis [2]. It usually causes extra-intestinal infections in patients with underlying chronic illnesses [5,11]. In addition, this organism also causes infection of the biliary tree [12], ensuing in the formation of an abscess [13], patients with an organ transplant [5] or postsurgical indwelling urinary catheter [13]. It has also been known to cause infection due to bacteremia originated from the genitourinary tract, with a male preponderance (71%).

In a study conducted by Gunthard and Pennekamp, various underlying conditions were attributed to causing H.alvei infection. The study revealed the development of H.alvei-UTI in one patient who underwent aortic valve replacement. Our patient had a history of mild aortic insufficiency with severe aortic stenosis along with prior admission for enterococcal endocarditis and was scheduled to undergo transcatheter aortic valve replacement.

H. alvei was found in the urogenital tract of a total of 12 urine samples that were collected from indwelling catheters (6), clean-catch urine (3), scrotal smears (2), and episiotomy wound (1) [12]. Our patient had a prolonged history of self-catheterization performed about four times a day. He also had a previous history of recurrent UTI, so there is a likelihood that H.alvei-induced UTI could have developed as a result of a new variant organism due to prolonged catheterization. 

H. alvei is not one of the common etiological causes of UTI, and only a handful of cases have been reported. Moriuchi and Trucksis [14] reported a case of Hafnia alvei-induced UTI in a 44-year-old female resistant to ticarcillin, clavulanic acid, piperacillin, ceftriaxone, ceftazidime, and treated with intravenous ciprofloxacin. The patient in the present case had a urine culture growth of H. alvei leading to the development of UTI. 

H. alvei shows resistance to first-generation cephalosporin and amoxicillin. It is also showing rapid resistance to second- and third-generation cephalosporins [11], as seen in our case. One of the most attributable reasons for resistance to the newer generation antibiotics especially cephalosporins is due to inducible Bush group 1 beta-lactamase (Ambler class C) produced by H. alvei such as seen with ceftazidime and ticarcillin [2]. One of the strains of H. alvei containing the ampC gene manifests 94% amino acid sequence identity to a plasmid-borne cephalosporinase of Klebsiella pneumoniae [15]. Our patient had a culture and sensitivity report that showed resistance to most beta-lactam antibiotics, including cefuroxime, ceftriaxone, and ceftazidime, along with intermediate susceptibility to piperacillin/tazobactam. He was managed with intravenous cefepime based on the sensitivity report.

## Conclusions

In conclusion, physicians should be wary of H. alvei-induced urosepsis in patients with long-term catheterization. Also, there should be more emphasis on appropriate treatment against H. alvei infection due to the emerging risk of antibiotic resistance. Given this patient's co-morbidities, namely, mild aortic insufficiency with severe aortic stenosis, the management of this patient for septic shock was difficult, which led to mortality. Owing to a dearth of literature on this rare Gram-negative bacteria, our case can be treated as a step towards an extensive search for the pathogenicity of H. alvei.
